# Polycystic ovary syndrome, insulin resistance, and bone health in Saudi women: a cross-sectional study

**DOI:** 10.1007/s11657-026-01707-8

**Published:** 2026-09-15

**Authors:** Mai Albaik, Fiona E. A. McGuigan, Ahmed E. Altyar, Jalaluddin A. Khan, Kristina E. Akesson

**Affiliations:** 1 Department of Chemistry, Preparatory Year Program, Batterjee Medical College, Jeddah, 21442 Saudi Arabia; 2 Center of Excellence for Osteoporosis Research, King Abdulaziz University, Jeddah, 21589 Saudi Arabia; 3 Department of Clinical Sciences Malmö, Clinical and Molecular Osteoporosis Research Unit, Lund University, Lund, Sweden; 4 Department of Orthopaedics, Skåne University Hospital, Malmö, Sweden; 5 Department of Pharmacy Practice, Faculty of Pharmacy, King Abdulaziz University, Jeddah, 21589 Saudi Arabia; 6 Department of Pharmaceutical Sciences, Pharmacy Program, Batterjee Medical College, Jeddah, 21442 Saudi Arabia; 7 Biochemistry Department, Faculty of Sciences, King Abdulaziz University, Jeddah, 21589 Saudi Arabia

**Keywords:** Polycystic ovary syndrome, Insulin resistance, Bone health, Saudi women, Metabolic burden, Hormonal dysregulation

## Abstract

**Summary:**

Insulin resistance is common in Saudi women with PCOS and linked to hormonal imbalances. While bone health differences were not significant, trends suggest obesity and vitamin D may influence future risk. Early detection and management of insulin resistance could help reduce long-term health complications in affected women.

**Background:**

Polycystic ovary syndrome (PCOS) is a common endocrine disorder characterized by hormonal, metabolic, and reproductive dysfunction. Insulin resistance (IR), a hallmark feature of PCOS, is associated with long-term health complications, including adverse effects on bone metabolism.

**Objective:**

This study aimed to explore the prevalence of IR in Saudi women with PCOS and investigate its associations with hormonal, metabolic, and bone health parameters.

**Methods:**

A cross-sectional study was conducted at the Center of Excellence for Osteoporosis Research, King Abdulaziz University, Jeddah, Saudi Arabia, between December 2018 and June 2019. A total of 144 participants were enrolled, comprising 72 women with PCOS and 72 age- and BMI-matched healthy controls. Anthropometric measurements, biochemical analysis, and bone mineral density (BMD) assessments were performed. IR was evaluated using fasting serum insulin, homeostasis model assessment (HOMA), and glucose-to-insulin ratio (GIR). Statistical analyses included group comparisons and correlation analyses to identify associations.

**Results:**

IR was significantly higher in women with PCOS compared to controls across all diagnostic criteria (*P* < 0.01). PCOS women had higher luteinizing hormone (LH) levels and LH/FSH ratios (*P* < 0.001), particularly in insulin-resistant subgroups. No significant differences in BMD or T-scores were observed between groups; however, stratified analyses suggested trends toward higher T-scores in obese PCOS women. Vitamin D deficiency was similarly prevalent in both groups.

**Conclusion:**

This study highlights the substantial metabolic burden of IR in Saudi women with PCOS and its association on hormonal profiles. While no differences in bone health were observed, trends suggest that factors such as BMI and vitamin D status may be associated with skeletal outcomes at later stages. These findings underscore the importance of early IR identification and management to mitigate long-term health complications in PCOS.

## Introduction

Polycystic ovary syndrome (PCOS) is a complex endocrine disorder affecting approximately 4–20% of women of reproductive age worldwide [1]. It is characterized by hyperandrogenism, ovulatory dysfunction, and polycystic ovaries, and is associated with various metabolic abnormalities, including insulin resistance (IR) [2, 3]. Insulin resistance is a key feature of PCOS, contributing to the pathogenesis of the syndrome and its associated complications, such as type 2 diabetes and cardiovascular disease [4].

In Saudi Arabia, PCOS is a significant health concern, with a reported prevalence of approximately16% [5]. Despite its high prevalence, there is a paucity of research investigating the specific impact of insulin resistance on endocrine, metabolic, and bone health parameters among Saudi women with PCOS. Addressing this gap is crucial for developing effective management strategies to improve the health outcomes of affected women in Saudi Arabia.

This study aims to address this gap in knowledge; firstly by reviewing the literature from the past decade to explore the interplay between PCOS, insulin resistance, and bone health in diverse populations, including those from the Middle East and other regions worldwide Secondly, by evaluating of insulin resistance in Saudi women diagnosed with polycystic ovary syndrome using three diagnostic criteria: fasting insulin, homeostasis model assessment (HOMA), and glucose-to-insulin ratio (GIR). The specific objectives of the study are (1) to evaluate endocrine and metabolic characteristics and bone health parameters in women with PCOS and their corresponding controls, stratified by BMI and IR status and (2) to explore the correlations between insulin resistance and these parameters in women with PCOS.

The ultimate purpose is to provide critical insights into the pathophysiological role of insulin resistance in PCOS and its implications for endocrine and metabolic health, particularly in the context of Saudi women.

## Subjects and methods

### Subjects and study design

The study was conducted at the Center of Excellence for Osteoporosis Research (CEOR), King Abdulaziz University (KAU), Jeddah, Saudi Arabia, from December 2018 to June 2019.

A total of 173 Saudi women were recruited for potential inclusion in the study.

Among them, women diagnosed with polycystic ovary syndrome (PCOS) were first identified and prospectively recruited during their visits to the Infertility Clinic at King Abdulaziz University Hospital. Eligible participants were then referred to the CEOR clinic for further assessment.

**Fig. 1:**
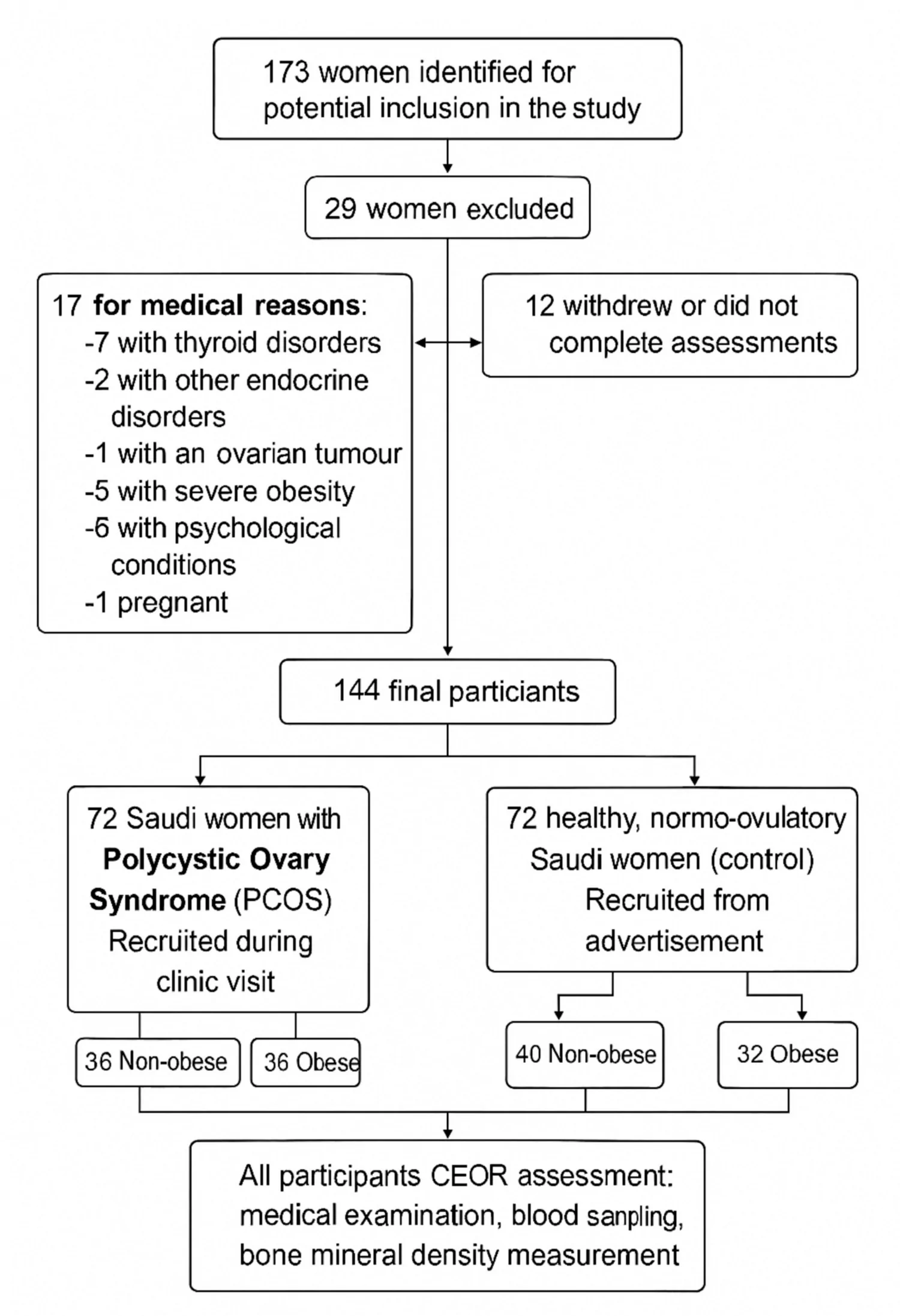
Sample Size and Participant Distribution

After applying exclusion criteria, 72 women with confirmed PCOS were included in the final analysis. Excluded cases (*n* = 29) involved women with thyroid disorders, other endocrine disorders, ovarian tumour, severe obesity, pregnancy, psychological conditions, or withdrawal of consent (Fig. 1).

For each PCOS participant, a matched control (normo-ovulatory, healthy Saudi woman) was selected based on age and body mass index (BMI). Control participants were recruited through public advertisements and underwent clinical screening at CEOR to confirm eligibility.

In total, the final cohort consisted of 144 participants: 72 women with PCOS and 72 matched controls. All participants underwent medical examinations, blood sampling, and bone mineral density (BMD) measurements at the CEOR clinic.

The study was approved by the Human Research Ethics Committee of CEOR, KAU, and adhered to the ethical standards of the Helsinki Declaration of 1975. Written informed consent was obtained from all participants.

The **literature review** included studies published between 2014 and 2024 covering topics related to PCOS, insulin resistance, and bone health. A comprehensive search was conducted in the PubMed database using the keywords “Polycystic Ovary Syndrome,” “Insulin Resistance,” and “Bone Health,” as well as combined terms such as “PCOS and Insulin Resistance” and “PCOS and Bone Health.” The search included studies from diverse populations worldwide, including those from the Middle East, as summarized in Table 1 [6–14].

**Table 1:** Summary of Recent Studies on PCOS, Insulin Resistance, and Bone Health (2014–2024)

SN	Cohort	Country	Year	ages	outcomes	Main results	References
1	**69** PCOS women & **30** healthy controls	Poland	2014	PCOS: 17–34 yrs Controls: 20–30 yrs	Bone mineral density (BMD) in the lumbar spine was measured using dual-energy X-ray absorptiometry (DXA)	✓ PCOS women had lower BMD values ✓ BMD was positively correlated with insulin concentration and HOMA–IR	[6]
2	**52** PCOS women & **39** healthy controls	China	2016	PCOS: 17–27 yrs Controls: 25–27 yrs	The study measured serum irisin levels, body composition (including body mass index (BMI) and total lean mass), bone mineral density, and adiponectin levels	✓ The abnormal body composition in PCOS may contribute to the circulation irisin ✓ The crosstalk of irisin in different organs was found and may be related to disease development in PCOS	[7]
3	**60** PCOS women & **60** healthy controls	Canda	2018	18–35 yrs	Measures of hip geometry, including cross-sectional area, cross-sectional moment of inertia, subperiosteal width (SPW), and section modulus, were compared between groups. Bone mineral density (BMD) at various sites was also measured	women with PCOS may have compromised intertrochanter SPW while oligomenorrhea appears to have no detrimental effect on bone density or geometry in women with PCOS	[8]
4	**60** PCOS women & **58** healthy controls	India	2018	14–24 yrs	Bone mineral density (BMD) was measured using dual energy X-ray absorptiometry	✓ **BMI** is the most important **determinant** of BMD in women with PCOS ✓ **BMD** is *not different between healthy young women and those with PCOS*	[9]
5	**80** PCOS women & **15** healthy controls	Egypt	2018	22–40 yrs	BMD of the spine and femur was assessed using DXA. Serum irisin level, fasting insulin, and HOMA-IR were measured	✓ Serum irisin level may be considered as a novel biomarker for PCOS diagnosis ✓ Circulating irisin in PCOS is strongly related to BMD ✓ Irisin may be associated with bone metabolism	[10]
6	**143** PCOS women & **60** healthy controls	USA	2020	PCOS: 22–29 yrs controls: 28–32 yrs	Skeletal muscle index% (SMI%) and BMD were evaluated in all groups using DXA	✓ PCOS women exhibit early signs of osteosarcopenia when compared to controls likely attributed to disrupted insulin function ✓ Understanding the degree of musculoskeletal deterioration in PCOS is critical for implementing targeted interventions that prevent and delay osteosarcopenia in this clinical population	[11]
7	**33** PCOS women & **32** healthy controls	India	2022	18–50 yrs	The association of anti-thyroid peroxidase antibodies (anti-TPO) with the gonadotropin profile (LH/FSH ratio) in PCOS women, along with other parameters	A high prevalence of autoimmune thyroiditis in euthyroid PCOS women and suggests a strong link of euthyroid obese PCOS women to autoimmunity due to the hyper-anderogenism and a higher LH/FSH ratio	[12]
8	**123** PCOS women (56 non-obese and 67 obese) & **45** normal weight controls	Turkey	2023	PCOS: 28.13 ± 4.43 yrs Controls: 26.53 ± 2.67 yrs	The relationship between BMD, vitamin D status, insulin resistance, sex hormones, and calcium metabolism disorders in women with PCOS	✓ PCOS women, especially those with obesity and hyperandrogenemia, often have vitamin D deficiency ✓ Non-obese PCOS women show lower BMD than healthy controls, but obese PCOS women have similar BMD to normal-weight eumenorrheic controls ✓ MBI is the primary factor influencing BMD in PCOS women	[13]
9	**61** PCOS women & **35** healthy controls	Iran	2023	18–41 yrs	Comparing PCOS women to controls to study hormonal effects on bone density, measuring BMC and BMD with DXA, and evaluating blood hormone levels and HOMA-IR	✓ BMI and HOMA-IR were independent and positively related to FN and total hip bone density in PCOS subjects ✓ However, in non-PCOS women, BMI was the sole independent determinant of bone density at most sites ✓ Osteocalcin showed an inverse correlation with BMI in both groups	[14]

The study was approved by the Human Research Ethics Committee of CEOR, KAU, and adhered to the ethical standards of the Helsinki Declaration of 1975. Written informed consent was obtained from all participants.

### Anthropometric measurements

Age, body weight, height, body mass index (BMI), and waist-to-hip ratio (WHR) were recorded. The BMI was calculated as weight/(height)^2^ in kilograms per square meter, and women were considered non-obese at BMI < 30 kg/m^2^; women were considered obese at BMI ≥ 30 kg/m^2^. The WHR was calculated as waist circumference in centimetres divided by hip circumference in centimetres. All women were medically examined and interviewed using a standardized questionnaire to collect information on medical history, anthropometric measurement, lifestyle, drug history, menstrual and obstetrical history.

### Biochemical analysis

Venous blood samples were collected in the morning following an 8-h fasting period. Serum samples were then centrifuged at 3,000 g for 15 min and stored at −80 °C until analysis. All analysis were performed at the Clinical Diagnostics Laboratory at CEOR including the reported intra- and inter-assay coefficients of variation.

Follicle-stimulating hormone (FSH), luteinizing hormone (LH), and glucose concentrations were measured using the VITROS 250 Chemistry System Autoanalyzer (Ortho-Clinical Diagnostics, Johnson & Johnson, USA). Serum 25-hydroxyvitamin D [25(OH)D] and intact parathyroid hormone (PTH) levels were analyzed using the LIASON autoanalyzer (DiaSorin Inc., Stillwater, MN, USA).

#### FSH

Intra- and inter-assay coefficients of variation (CVs) were both < 4.0%. The functional sensitivity of the assay was 0.66 IU/L, according to the manufacturer’s information sheet. Results are expressed in IU/L.

#### LH

Intra- and inter-assay CVs were both < 4.0%. The assay’s functional sensitivity was 0.216 IU/L, based on the manufacturer’s specifications. Results are expressed in IU/L.

#### Glucose

Intra- and inter-assay CVs were 0.05% and 0.15%, respectively. Results are expressed in mmol/L. Measurements were made using VITROS GLU Slides and the VITROS Chemistry Products Calibrator Kit 1 on VITROS Chemistry Systems.

#### Insulin

Insulin levels were measured using kits from DiaSorin, Italy. The intra- and inter-assay CVs were 3.0% and 4.7%, respectively. The functional sensitivity ranged from 0.51 to 0.87 μU/mL, according to the manufacturer’s specifications. Results are expressed in μU/mL.

#### Vitamin D [25(OH)D]

The intra- and inter-assay CVs for serum [25(OH)D] were 7.8% and 3.8%, respectively. The assay’s functional sensitivity was ≤ 9.984 nmol/L, based on the manufacturer’s information. Results are expressed in nmol/L.

#### Serum intact PTH

Intra- and inter-assay CVs for intact PTH were 5.1% and 4.3%, respectively. The functional sensitivity of the assay was ≤ 0.106 pmol/L, as indicated by the manufacturer’s information. Results are expressed in pmol/L.

### Insulin resistance

In this study, insulin resistance (IR) was assessed using three criteria: (1) fasting serum insulin, (2) HOMA, and (3) glucose-to-insulin ratio (GIR). Fasting serum insulin levels were measured, with values exceeding the 95th percentile of healthy, normo-ovulatory, non-obese control women considered indicative of IR.

HOMA was calculated using the formula: fasting plasma glucose (mmol/L) × fasting serum insulin (μU/mL)/22.5, with higher scores indicating IR.

The GIR, proposed as an index of peripheral insulin sensitivity, was also calculated as the ratio of fasting glucose (mmol/L) to fasting insulin (pmol/L). GIR provides a direct, quantitative assessment that is more sensitive and reliable than surrogate indices, particularly for detecting early pharmacological improvements [15]. A cutoff value of GIR < 4.5 was applied to define insulin resistance, consistent with previously validated thresholds and prior studies in obese women and women with PCOS [16, 17]. Values below this level reliably indicate a physiologically meaningful reduction in insulin‑stimulated glucose disposal accompanied by incomplete suppression of hepatic glucose output [18, 19].

Insulin resistance was defined as an abnormal result in any of the three criteria: fasting insulin > 64.4 pmol/L, HOMA > 3.82 or GIR < 4.5, based on thresholds established from the control group [16]. For the purpose of classification and comparison between groups, GIR was used as the primary indicator of insulin resistance.

### Dual-energy X-ray absorptiometry (DXA)

Dual-Energy X-ray Absorptiometry (DXA) was used to assess bone mineral density (BMD, g/cm^2^) and T-score using the GE Healthcare Lunar iDXA system (USA). T-scores were automatically calculated by the DXA system using standard reference settings, generally consistent with the NHANES III White female population, in accordance with the recommendations of the International Osteoporosis Foundation (IOF) and the International Society for Clinical Densitometry (ISCD), as described by Kanis et al. [20]. Periodic and daily precision tests were performed following the manufacturer’s guidelines, with a mean precision error of 1.1% for the lumbar spine and 0.7% for the total hip. Bone status was classified according to World Health Organization (WHO) criteria: normal (T-score >  − 1), osteopenia (T-score − 1 to − 2.5), and osteoporosis (T-score <  − 2.5) [21].

### Statistical analysis

To compare differences between the PCOS group and the control group, as well as between subgroups (non-obese and obese, and IR and non-IR), an independent t-test was applied for continuous variables, which were expressed as mean ± standard deviation (SD).

Insulin resistance was primarily classified according to the glucose-to-insulin ratio (GIR < 4.5), which served as the main indicator for statistical comparisons between IR and non-IR subgroups.

For categorical variables, the chi-square test was used. Prevalence and incidence rates (IR) were presented as percentages.

Given the high prevalence of vitamin D deficiency across both PCOS and control groups, vitamin D was not included as an adjustment variable in the primary analyses of bone outcomes. All reported comparisons and associations are unadjusted. This approach was adopted because vitamin D levels did not differ significantly between groups and therefore were unlikely to confound between-group comparisons.

The statistical analysis was conducted using IBM® SPSS® version 26. Normality of continuous variables was assessed using the Shapiro–Wilk test. Variables that deviated from normal distribution were log-transformed prior to analysis. Given the exploratory nature of the study, no formal correction for multiple comparisons was applied. All statistical tests were considered nominally significant at *p*-value < 0.05.

## Results

The mean age was 28.9 (7.1) years, range 24–44 years. Women with PCO had a BMI of 30.3 with 36/72 (50%) women classified as obese (BMI > 30) and controls had a BMI of 28.7 with 32/72 (44.4%) classified as obese (Table 2).

**Table 2:** Age and anthropometric data by BMI (Non-obese vs Obese)

Variables	All women (*n* = 144)			BMI: Non-obese and Obese					
	PCOS (*n* = 72)	Controls (*n* = 72)	*P*- value	PCOS Non-obese (*n* = 36)	Control Non-obese (*n* = 40)	*P*- value	PCOS Obese (*n* = 36)	Controls Obese (*n* = 32)	*P*- value
Age (year)	28.9 ± 7.1	28.9 ± 6.6	0.919	28.5 ± 7.5	26.5 ± 5.4	0.460	29.4 ± 6.8	31.9 ± 6.8	0.211
Weight (kg)	73.8 ± 20.8	70.6 ± 15.0	0.447	56.8 ± 11.6	60.2 ± 9.1	0.323	90.9 ± 11.9	83.6 ± 9.6	0.059
BMI (kg/m^2^)	30.3 ± 8.3	28.7 ± 6.7	0.387	1.6 ± 0.1	1.6 ± 0.1	0.176	1.6 ± 0.1	1.6 ± 0.0	0.905
HC (cm)	105.0 ± 14.2	103.0 ± 13.6	0.549	23.1 ± 3.4	24.0 ± 3.7	0.407	37.5 ± 4.5	34.6 ± 4.6	0.075
WC (cm)	84.5 ± 15.6	80.6 ± 13.0	0.255	92.9 ± 5.9	94.3 ± 8.6	0.594	117.1 ± 8.5	114.0 ± 10.2	0.347
WHR	0.80 ± 0.07	0.78 ± 0.06	0.236	71.9 ± 9.7	73.1 ± 9.1	0.707	97.0 ± 8.6	89.9 ± 11.1	**0.045**

Values are presented as means ± SD; *PCOS* polycystic ovary syndrome, *BMI* body mass index, Non-obese (< 30 kg/cm2) and Obese (≥ 30 kg/m2), *HC* hip circumference, *WC* waist circumference, *WHR* waist to hip ratio

### Prevalence of insulin resistance

**Fig. 2:**
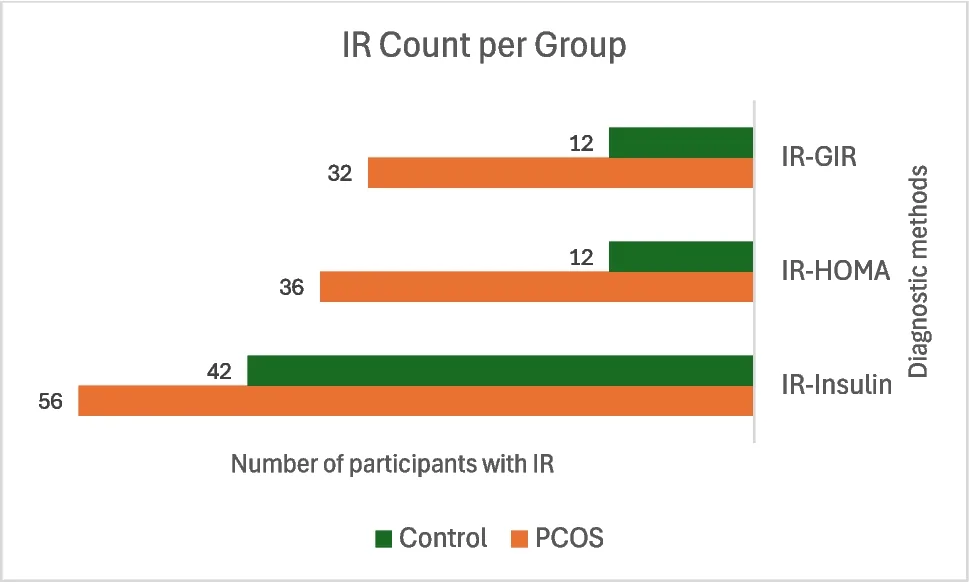
Number of participants with insulin resistance (IR) among women with PCOS and controls

Figure 2 shows the prevalence of insulin resistance (IR) among PCOS and control participants. Participants were classified as IR or non-IR based on the following criteria: fasting insulin > 64.4 pmol/L, HOMA > 3.82 or GIR < 4.5.

Using fasting insulin, IR was observed in 56 of 72 (77.8%) PCOS women vs 42 of 72 (58.3%) controls. By HOMA-IR, 36 of 72 (50%) PCOS women were IR compared to 12 of 72 (16.7%) controls. GIR analysis showed 32 of 72 (44.4%) PCOS women vs 12 of 72 (16.7%) controls (*P* < 0.01 for all comparisons).

### Anthropometric data

The anthropometric characteristics of the study population are summarized in Table 2. There were no statistically significant differences in BMI, waist circumference (WC), or hip circumference (HC) between PCOS and control groups. However, waist-to-hip ratio (WHR) was significantly higher in obese PCOS women (0.84 ± 0.1) compared to their obese controls (0.79 ± 0.1; *P* < 0.05). Table 3 shows stratified data by IR, revealing a trend toward higher WHR women with PCOS and IR.

**Table 3:** Age and anthropometric data by IR classification (GIR < 4.5 vs ≥ 4.5) in women with PCOS and controls and in PCOS Non-obese and Obese

Variables	IR: GIR < 4.5 and Non-IR: GIR ≥ 4.5						Non-obese (*n* = 36)			Obese (*n* = 36)		
	PCOS Non-IR (*n* = 40)	Control Non-IR (*n* = 60)	P-value	PCOS IR (*n* = 32)	Control IR (*n* = 12)	*P*-value	PCOS Non-IR (*n* = 28)	PCOS IR (*n* = 8)	*P*- value	PCOS Non-IR (*n* = 12)	PCOS IR (*n* = 24)	*P*- value
Age (year)	30.1 ± 8.0	29.3 ± 6.9	0.734	27.6 ± 5.5	27.0 ± 4.9	0.822	29.1 ± 7.6	26.3 ± 7.2	0.517	32.17 ± 9.1	28.0 ± 5.1	0.339
Weight (kg)	63.8 ± 18.6	69.9 ± 13.1	0.207	86.4 ± 16.3	73.7 ± 23.7	0.265	53.5 ± 9.5	67.0 ± 13.7	0.150	86.8 ± 12.8	92.8 ± 11.3	0.355
BMI (kg/m^2^)	26.5 ± 8.0	28.4 ± 5.7	0.372	35.0 ± 6.1	30.5 ± 11	0.380	22.1 ± 2.9	26.2 ± 2.8	0.054	36.6 ± 6.4	37.9 ± 3.4	0.675
HC (cm)	99.2 ± 12.5	102.6 ± 12.3	0.337	112.3 ± 13	105 ± 20.1	0.436	92.2 ± 5.49	95.5 ± 7.59	0.465	115.3 ± 8.1	117.9 ± 8.8	0.549
WC (cm)	76.8 ± 13.3	80 ± 11.2	0.379	94.1 ± 12.8	83.7 ± 21.3	0.300	70.7 ± 9.84	76.2 ± 8.95	0.333	90.8 ± 9.1	100.0 ± 6.7	0.059
WHR	0.77 ± 0.1	0.78 ± 0.1	0.669	0.84 ± 0.1	0.79 ± 0.1	0.200	0.76 ± 0.07	0.79 ± 0.03	0.247	0.78 ± 0.03	0.85 ± 0.07	**0.021**

Values are presented as means ± SD; *PCOS* polycystic ovary syndrome, *BMI* body mass index, *HC* hip circumference, *WC* waist circumference, *WHR* waist to hip ratio, *IR* insulin resistance, *GIR* glucose to insulin ratio

### Endocrine and metabolic characteristics

Table 4 outlines the endocrine and metabolic profiles of PCOS and control women. Women with PCOS exhibited significantly higher LH levels compared to controls (9.4 ± 8.6 vs. 4.1 ± 3.7 IU/L; *P* < 0.001). The LH/FSH ratio was also markedly higher in the PCOS group (*P* < 0.001), with pronounced differences in women who were non-obese and IR (Table 5).

**Table 4:** Endocrine and metabolic analyses by BMI (non-obese; obese)

Variables	All women (*n* = 144)			BMI: Non-obese and Obese					
	PCOS (*n* = 72)	Control (*n* = 72)	*P*- value	PCOS Non-obese (*n* = 36)	Control Non-obese (*n* = 40)	*P*- value	PCOS Obese (*n* = 36)	Control Obese (*n* = 32)	*P*- value
FSH (IU/L)	5.8 ± 5.1	5.0 ± 2.8	0.822	6.7 ± 6.5	5.2 ± 3.3	0.613	5.0 ± 3.1	4.6 ± 2.0	0.798
LH (IU/L)	9.4 ± 8.6	4.1 ± 3.7	**0.0001**	12.2 ± 11.1	4.2 ± 3.0	**0.002**	6.6 ± 3.3	3.8 ± 4.5	**0.002**
LH/FSH	2.0 ± 1.4	0.8 ± 0.7	**0.0001**	2.3 ± 1.5	0.9 ± 0.6	**0.0001**	1.7 ± 1.4	0.8 ± 0.8	**0.003**
Fasting glucose (mmol/)	4.8 ± 1.0	4.6 ± 0.5	0.782	4.8 ± 1.0	4.6 ± 0.5	0.782	4.3 ± 0.4	4.5 ± 0.4	0.482
Fasting insulin (μU/ml)	21.2 ± 14.8	11.9 ± 6.5	**0.005**	21.2 ± 14.8	11.9 ± 6.5	**0.005**	14.2 ± 11.0	11.1 ± 6.4	0.613
HOMA	4.7 ± 3.9	2.5 ± 1.5	**0.004**	4.7 ± 3.9	2.5 ± 1.5	**0.004**	2.7 ± 2.1	2.2 ± 1.3	0.593
GIR	7.1 ± 6.7	9.3 ± 5.3	**0.008**	7.1 ± 6.7	9.3 ± 5.3	**0.008**	9.2 ± 8.0	10.3 ± 6.0	0.515
25(OH)D (nmol/)	21.1 ± 15.1	22.0 ± 19.5	0.735	21.2 ± 17.5	27.3 ± 24.7	0.443	21.0 ± 12.7	15.3 ± 5.0	0.175
Intact-PTH (pmol/)	11.0 ± 7.9	12.2 ± 6.4	0.265	9.0 ± 4.3	10.5 ± 6.5	0.633	13.0 ± 10.1	14.3 ± 5.9	0.224

Values are presented as means with their standard deviations. PCOS: polycystic ovary syndrome; *BMI* body mass index, Non-obese (< 30 kg/cm2) and Obese (≥ 30 kg/m2), *FSH* follicular-stimulating hormone, *LH* luteinizing hormone, *HOMA* homeostasis model assessment, *GIR* glucose to insulin ratio, *PTH* parathyroid hormone

**Table 5:** Endocrine and metabolic data by IR classification (GIR < 4.5 vs ≥ 4.5) in women with PCOS and controls and in PCOS Non-obese and Obese

Variables	IR: GIR < 4.5 and Non-IR: GIR ≥ 4.5						Non-obese (*n* = 36)			Obese (*n* = 36)		
	PCOS Non-IR (*n* = 40)	Control Non-IR (*n* = 60)	*P*-value	PCOS IR (*n* = 32)	Control IR (*n* = 12)	*P*-value	PCOS Non-IR (*n* = 28)	PCOS IR (*n* = 8)	*P*- value	PCOS Non-IR (*n* = 12)	PCOS IR (*n* = 24)	*P*- value
FSH (IU/L)	6.7 ± 6.1	4.7 ± 2.3	0.179	4.7 ± 3.2	6.0 ± 4.7	0.543	7.9 ± 6.8	2.0 ± 0.3	**0.006**	3.8 ± 2.4	5.5 ± 3.2	0.214
LH (IU/L)	12.2 ± 10.4	3.9 ± 3.9	**0.002**	5.9 ± 3.5	4.6 ± 2.8	0.420	14.9 ± 11.2	2.7 ± 2.7	**0.001**	5.7 ± 3.2	7.0 ± 3.3	0.456
LH/FSH	2.5 ± 1.6	0.8 ± 0.6	**0.0001**	1.4 ± 0.8	1.0 ± 0.9	0.303	2.5 ± 1.5	1.4 ± 0.6	0. 055	2.2 ± 2.0	1.4 ± 0.8	0.364
Fasting glucose (mmol/)	4.7 ± 1.2	4.6 ± 0.5	0.504	4.9 ± 0.9	4.8 ± 0.5	0.654	4.3 ± 0.4	4.1 ± 0.1	0.306	5.7 ± 1.7	5.2 ± 0.8	0.517
Fasting insulin (μU/ml)	10.5 ± 4.6	9.8 ± 4.8	0657	34.6 ± 11.8	22.4 ± 2.5	**0.022**	9.1 ± 3.8	31.8 ± 9.3	**0.014**	13.6 ± 5.0	35.6 ± 12.7	**0.0001**
HOMA	2.3 ± 1.4	2.0 ± 1.1	0.497	7.8 ± 3.9	4.8 ± 0.9	**0.009**	1.7 ± 0.8	5.8 ± 1.8	**0.017**	3.4 ± 0.7	8.4 ± 1.2	**0.003**
GIR	10.6 ± 7.4	10.4 ± 5.1	0.952	2.7 ± 0.7	3.9 ± 0.4	**0.0001**	11.1 ± 8.0	2.5 ± 0.7	**0.001**	9.2 ± 2.4	2.8 ± 0.2	0.047
25(OH)D (nmol/)	17.9 ± 7.1	20.1 ± 15.3	0.505	25.2 ± 20.8	31.4 ± 34.3	0.688	17.5 ± 7.1	33.9 ± 35.1	0.422	1.8 ± 7.6	2.2 ± 14.7	0.508
intact-PTH (pmol/)	11.1 ± 5.3	11.9 ± 6.7	0.616	10.9 ± 10.5	13.4 ± 5.2	0.478	9.9 ± 4.3	5.8 ± 1.8	0.016	13.8 ± 6.7	12.6 ± 11.7	0.797

Values are presented as means with their standard deviations. *PCOS* polycystic ovary syndrome, *BMI* body mass index, *IR* insulin resistance, *FSH* follicular-stimulating hormone, *LH* luteinizing hormone, *HOMA* homeostasis model assessment, *GIR* glucose to insulin ratio, *PTH* parathyroid hormone

PCOS women classified as IR, had significantly higher fasting serum insulin levels compared to IR controls (34.6 ± 11.8 vs. 22.4 ± 2.5 µU/mL; *P* < 0.05), higher HOMA values (*P* < 0.01) and lower GIR values (*P* < 0.001), indicative of more severe insulin resistance.

Vitamin D (25(OH)D) levels were similarly low across both PCOS and controls (21.1 ± 15.1 vs. 22.0 ± 19.5 nmol/L; *P* = 0.735). Parathyroid hormone (PTH) levels were also comparable between groups (PCOS 11.0 ± 7.9 pmol/L vs 12.2 ± 6.4 pmol/L; *P* = 0.265).

### Bone mineral density (BMD) and T-scores

The bone health parameters, including BMD and T-scores, are presented in Tables 6 and 7. No statistically significant differences were noted in BMD or T-scores at the lumbar spine (L1-L4) or femoral neck between PCOS women and controls. However, a non-significant trend toward higher T-scores in obese PCOS women was noted.

**Table 6:** Bone mineral density by BMI (non-obese; obese)

Variables		All women (*n* = 144)			BMI: Non-obese and Obese					
		PCOS (*n* = 72)	Control (*n* = 72)	*P*- value	PCOS Non-obese (*n* = 36)	Control Non-obese (*n* = 40)	*P*- value	PCOS Obese (*n* = 36)	Control Obese (*n* = 32)	*P*- value
Spine (L1-L4)	BMD g/cm^2^	1.139 ± 0.116	1.131 ± 0.0878	0.753	1.117 ± 0.120	1.138 ± 0.087	0.550	1.161 ± 0.111	1.123 ± 0.091	0.283
	T-score	0.29 ± 1.0	0.24 ± 0.7	0.806	0.067 ± 1.045	0.270 ± 0.698	0.491	0.506 ± 0.936	0.194 ± 0.696	0276
Femur Neck	BMD g/cm^2^	0.949 ± 0.115	0.955 ± 0.104	0.826	0.893 ± 0.114	0.950 ± 0.122	0.146	1.006 ± 0.086	0.961 ± 0.079	0.129
	T-score	0.14 ± 1.0	0.21 ± 0.8	0.754	−0.306 ± 0.934	0.150 ± 1.021	0.160	0.589 ± 0.763	0.281 ± 0.551	0.132

Values are presented as mean ± SD; PCOS: polycystic ovary syndrome; BMI: body mass index, Non-obese (< 30 kg/cm2) and Obese (≥ 30 kg/m2), *BMD* body bone density. T‑scores were calculated using the NHANES III White female reference population according to ISCD guidelines (Kanis JA et al., Osteoporos Int 2013). Osteopenia: T‑score –1.0 to –2.5; osteoporosis: T‑score ≤ –2.5

**Table 7:** Bone mineral density by IR classification (GIR < 4.5 vs ≥ 4.5) in women with PCOS and controls and in PCOS Non-obese and Obese

Variables		IR: GIR < 4.5 and Non-IR: GIR ≥ 4.5						Non-obese (*n* = 36)			Obese (*n* = 36)		
		PCOS Non-IR (*n* = 40)	Control Non-IR (*n* = 60)	P-value	PCOS IR (*n* = 32)	Control IR (*n* = 12)	*P*-value	PCOS Non-IR (*n* = 28)	PCOS IR (*n* = 8)	*P*- value	PCOS Non-IR (*n* = 12)	PCOS IR (*n* = 24)	*P*- value
Spine (L1-L4)	BMD g/cm^2^	1.13 ± 0.1	1.14 ± 0.1	0.533	1.16 ± 0.1	1.07 ± 0.1	0.065	1.118 ± 0.119	1.110 ± 0.142	0.923	1.142 ± 0.068	1.170 ± 0.123	0.555
	T-score	0.14 ± 1.0	0.31 ± 0.7	0.478	0.48 ± 1.1	−0.13 ± 0.5	0.085	0.050 ± 1.05	0.125 ± 1.178	0.914	0.333 ± 0.564	0.592 ± 1.089	0.517
Femur Neck	BMD g/cm^2^	0.91 ± 0.1	0.96 ± 0.1	0.132	1.00 ± 0.1	0.93 ± 0.1	0.221	0.883 ± 0.118	0.928 ± 0.107	0.506	0.976 ± 0.059	1.021 ± 0.096	0.236
	T-score	−0.21 ± 0.9	0.24 ± 0.8	0.093	0.56 ± 0.8	0.05 ± 0.8	0.220	−0.450 ± 0.968	0.200 ± 0.668	0.231	0.367 ± 0.508	0.700 ± 0.861	0.319

Values are presented as mean ± SD; *PCOS* polycystic ovary syndrome, *BMI* body mass index, *IR* insulin resistance, *GIR* glucose to insulin ratio, *BMD* body bone density. T‑scores were calculated using the NHANES III White female reference population according to ISCD guidelines (Kanis JA et al., Osteoporos Int 2013). Osteopenia: T‑score –1.0 to –2.5; osteoporosis: T‑score ≤ –2.5

### Correlation analysis

In women with PCOS, correlation analysis revealed significant associations between IR and hormonal parameters. GIR scores positively correlated with LH levels (*r* = 0.45, *P* < 0.01) and the LH/FSH ratio (*r* = 0.38, *P* < 0.05). Conversely, no significant correlations were identified between IR and bone health parameters, including BMD and T-scores, across the study population.

## Discussion

This study provides insights into the complex interplay between polycystic ovary syndrome (PCOS), insulin resistance, and bone health in Saudi women. Our findings confirm a significantly higher prevalence of IR in women with PCOS compared to controls, consistent across all diagnostic criteria; fasting insulin, homeostasis model assessment (HOMA), and glucose-to-insulin ratio (GIR). This aligns with prior studies indicating that IR is strongly associated with metabolic and hormonal dysregulation in PCOS. It is noteworthy that differences in IR prevalence across the three methods may stem from variations in their accuracy and sensitivity, with fasting insulin potentially overestimating IR, HOMA sometimes underestimating it, and GIR providing a more direct assessment of peripheral insulin sensitivity [3, 16].

### Hormonal and metabolic dysregulation in PCOS

The higher luteinizing hormone (LH) levels and LH/FSH ratios observed in the PCOS cohort reflect the characteristic hormonal disturbances associated with the syndrome. These findings are in line with prior research suggesting that hyperinsulinemia exacerbates gonadotropin dysregulation, leading to increased ovarian androgen production [22, 23]. Notably, the differences were more pronounced in insulin-resistant women, highlighting the synergistic effects of IR on endocrine dysfunction. The significant correlation between GIR scores and both LH levels and the LH/FSH ratio further underscores the role of IR in amplifying hormonal imbalances.

### Bone health in women with PCOS

Despite profound metabolic and hormonal alterations, our study did not identify differences in bone mineral density (BMD) or T-scores between women with PCOS and controls. These findings are consistent with studies such as McBreairty et al., which reported comparable bone geometry and density in PCOS and control populations [24]. However, stratified analyses showed a trend toward higher T-scores in obese PCOS women, suggesting that adiposity may exert a protective effect on bone health. The influence by body weight on BMD is well known, however, obesity’s influence on bone metabolism, possibly mediated by increased mechanical loading and adipokine secretion, may warrant further exploration in women with PCOS and associated metabolic disturbances [6, 25].

### Vitamin D deficiency and bone health

Vitamin D deficiency was prevalent across the entire study population, reflecting regional trends of widespread hypovitaminosis D in the Middle East [26, 27]. Although no significant differences in vitamin D levels were observed between groups, insulin-resistant PCOS women appeared to have tendency toward higher serum 25(OH)D levels, albeit non-significant. A larger study may allow for further insight into potential compensatory mechanisms or variations in vitamin D metabolism linked to IR and obesity, as previously suggested in the literature [6, 10, 13, 14].

Given the absence of significant between-group differences in serum 25(OH)D concentrations, vitamin D was not included as a covariate in the analyses of bone outcomes. While this approach reduces the likelihood of confounding in group comparisons, the possibility of residual or individual-level effects of vitamin D on bone health cannot be entirely excluded. Future studies employing larger sample sizes, adjusted analytical models, or longitudinal designs are therefore recommended to better elucidate the role of vitamin D in bone health among women with PCOS.

### Implications for clinical practice

The absence of significant correlations between IR and bone health parameters in this study is noteworthy. While IR has been implicated in impaired bone quality and reduced bone strength in other populations [6, 11], our findings suggest that additional factors, such as BMI and the hormonal environment, may play more prominent roles in shaping bone health outcomes in Saudi women with PCOS. These observations underscore the need for comprehensive approaches that address both metabolic and skeletal health in this population.

### Study limitations and future directions

This study’s cross-sectional design limits the ability to infer causality between IR and bone health outcomes. Accordingly, we have explicitly acknowledged this limitation and emphasized the need for longitudinal studies to clarify temporal and causal relationships between IR and skeletal outcomes. Additionally, the relatively small sample size and focus on a single ethnic group may constrain the generalizability of the findings. Future longitudinal studies with larger, more diverse cohorts are essential to unravel the temporal relationships between IR, hormonal dysregulation, and bone health in PCOS. Interventional studies targeting IR through lifestyle modifications or pharmacological therapies may also provide critical insights into mitigating its adverse effects on metabolic and skeletal health.

This study highlights the significant metabolic burden of insulin resistance in Saudi women with PCOS and its association with hormonal dysregulation. While no substantial differences in bone health were observed, trends suggest that factors such as BMI and vitamin D status may modulate skeletal outcomes in this population. Early identification and management of IR together with obesity are crucial for improving the long-term health of women with PCOS, particularly in regions with high prevalence of metabolic and endocrine disorders.

## Conclusion

This study demonstrates that insulin resistance is more frequently observed among Saudi women with PCOS, reflecting a substantial metabolic burden within this population. Insulin resistance was associated with more pronounced hormonal alterations, including higher LH levels and LH/FSH ratio. In contrast, no significant associations were observed between insulin resistance and bone health parameters at this age. Collectively, these findings underscore the importance of early identification and clinical monitoring of metabolic abnormalities in women with PCOS.

## Data Availability

No data availability statement is applicable for this article.
